# Guidance materials from 2014 to 2019 on nutritional care for Ebola patients in Ebola Treatment Units: an analysis

**DOI:** 10.1017/S136898002000261X

**Published:** 2021-01

**Authors:** Mija Ververs, Cecilia Vorfeld

**Affiliations:** 1 Emergency Response and Recovery Branch, Division of Global Health Protection, Center for Global Health, Centers for Disease Control and Prevention, 2500 Century Boulevard NE, Mailstop E-22, Atlanta 30345, GA, USA; 2 Medicine, Science and Humanities, Johns Hopkins University, Baltimore, MD, USA

**Keywords:** Ebola, Nutrition, Nutritional care, Ebola Treatment Unit, Guidance material

## Abstract

**Objective:**

To evaluate the inclusion and exclusion of nutritional content in guidance materials related to nutritional care for hospitalised Ebola Virus Disease (EVD) patients of any age with the aim to provide recommendations for future revised nutritional care guidelines in Ebola Treatment Units (ETU).

**Design:**

Qualitative and quantitative analyses of ETU protocols and other guidance materials were conducted. Materials were obtained from practitioners, their organisations and governments active in EVD outbreaks since 2014.

**Setting:**

Guinea, Liberia, Sierra Leone and Democratic Republic of Congo.

**Results:**

Guidance materials showed a wide variety of topics. Most contained information on different feeding phases during illness, the use of specialised products, what and how to feed children aged 0–23 months, and meal and snack frequency for different age groups. Most materials lacked guidance on how to assess or accommodate patients’ dietary preferences, how to obtain feedback on nutritional care from patients or how to assess whether patients need feeding support. These aspects are particularly relevant to prevent deterioration of the patients’ nutritional status. There was limited guidance on operational aspects of food preparation and provision.

**Conclusions:**

Since 2014, numerous materials have been developed by organisations and governments on nutritional support in ETU. Although every EVD outbreak response must be contextualised because of the complexity of EVD and its case management, it is important to resolve technical differences and to provide comprehensive and more practical guidance. The findings of this study may inform future revised guidelines from normative UN organisations and governments of countries affected by EVD.

On 1 August 2018, the Ministry of Health of the Democratic Republic of Congo reported a new outbreak of Ebola virus disease (EVD). This is the 10th and largest Ebola outbreak in Democratic Republic of Congo, and the second-largest outbreak of Ebola ever recorded since the virus was discovered in 1976 in Democratic Republic of Congo^(1)^. In 2019, a review was conducted on experiences and lessons learned from practitioners on the operational aspects of nutritional care and support in Ebola Treatment Units (ETU) in the 2014–2016 West Africa and current Democratic Republic of Congo EVD outbreak with the ultimate aim to improve current and future responses^(2)^. The review also evaluated the use and usefulness of the 2014 Interim Guidelines on providing nutritional support to patients in ETU that were issued by the WHO, the UN Children’s Fund (UNICEF) and the World Food Programme in November 2014^(3)^. These guidelines aimed to address nutritional needs and optimal nutritional care in the current Ebola crisis, with a particular focus on the practical aspects of the care within ETU for EVD patients. One of the reviews’ findings was that while the technical aspects of the 2014 Interim Guidelines were acceptable, its feasibility was questioned as it did not provide sufficient practical applications.

Since 2014, various non-governmental organisations, the Red Cross/Red Crescent Movement, UN agencies and governments have been directly involved in the care for EVD patients in the ETU. Many developed their own EVD outbreak nutritional care protocols and/or other guidance materials identifying best practices on the provision of food, including the method of preparation and distribution, what to provide to patients and how. Some materials addressed what was lacking in the 2014 Interim Guidelines and/or contextualised its guidance to their place of work. This study evaluates what topics were present or absent in guidance materials related to nutritional care for hospitalised EVD patients of any age. The aim is to provide recommendations on which topics to include in future revised guidelines on nutritional care in ETU from normative UN organisations and governments.

## Methodology

This study took place in June and July 2019 and examined guidance materials retrieved from practitioners, their organisations and governments that are or have been active in the EVD outbreaks.

Practitioners engaged in the nutritional care in the ETU and who participated in a previous investigation^(2)^ were approached to share their materials they used, which were developed to guide nutritional care in ETU by their respective organisations or by others (see Ref. (2) how practitioners were selected). Guidance materials that were stand-alone documents or part of larger protocols were included as long as they aimed to provide guidance on nutritional care. In addition, publicly accessible guidance materials were also evaluated. An initial screening of all materials by the authors resulted in a list of most common themes and sub-themes of all instructions found in the guidance materials. A second, more detailed analysis scored each document to indicate whether any instruction on a given theme was present or absent. Mentioning the sub-theme was sufficient to get a ‘present’ score, even if instructions lacked details. Scores were initially assigned by one author (C.V.) and independently verified by another (M.V.) author. Discrepancies were discussed and resolved. As scientific evidence on the best nutritional care in EVD patients is limited and lacks consensus^(4)^, no analysis was conducted to evaluate the accuracy of the instructions.

## Results

Eighteen different documents were retrieved^(3,5–21)^: three in French^(16–18)^ and fifteen in English. Only three were publicly available on the Internet^(3,20,21)^. Eight were from non-governmental organisations and the Red Cross/Red Crescent Movement^(5–12)^ of which one was co-authored by two different organisations^(11)^. Six were from governments (mostly Ministries of Health) with or without support from the UN^(13–18)^, and four were from UN agencies (WHO, UNICEF and World Food Programme)^(3,19–21)^. One guidance document was removed from analysis as it did not specifically refer to nutritional care in ETU^(15)^. Table 1 presents the list of the organisations and governments and the included materials.

**Table 1:** List of organisations and governments that provided guidance materials on nutritional support for Ebola patients in Ebola treatment units

	Number of documents provided
Non-governmental organisations	
GOAL International	1
International Medical Corps (IMC)	1
Médecins Sans Frontières (MSF)	1
Partners in Health	1
Save the Children	1
Red Cross/Red Crescent Movement	
International Committee of the Red Cross (ICRC)	1
International Federation of Red Cross and Red Crescent Societies (IFRC)	1
Governments	
Government of Sierra Leone	1
Ministry of Health and Social Welfare Republic of Liberia	1
République Démocratique du Congo Ministère de la Santé Publique	3
United Nations (UN) organisations	
United Nations Children’s Fund (UNICEF)	1
World Health Organization (WHO)	2
World Health Organization (WHO), United Nations Children’s Fund (UNICEF), World Food Programme (WFP) (co-authored)	1
Other	
International Committee of the Red Cross (ICRC) and Médecins Sans Frontières (MSF) (co-authored)	1
Total	17

Table 2 summarizes a list of all instruction topics found in the guidance materials and a percentage of guidelines where these topics were included (see online supplementary material, Supplemental Table for detailed results).

**Table 2:** Topics listed in guidance materials on nutritional care for Ebola patients in Ebola treatment units and the frequency of topic inclusion^(3,5–14,16–21)^

Nutritional Care Guidance Materials for Ebola Patients in Ebola Treatment Units	No. mentioning this topic (*n* 17)	%
Validity of guidance document		
Date/year of publication	14	82
Contains a statement of validity of document and its recommendations	1	6
Purpose of guidance document		
Clarification on the principles of treatment of EVD in relation to nutritional care and needs	10	59
EVD and feeding phases/groups		
Description of disease and feeding phases		
a) Affecting nutritional intake	11	65
b) Causing specific nutritional needs	10	59
c) Different feeding phases (maintenance, boost, transitions)	12	71
Description of different feeding groups (consistency: solid, semi-solid and liquid)	11	65
Assessment of patients with Ebola virus disease		
Instructions on whether and how to assess a patient’s dietary needs		
a) Pregnant and lactating women	2	12
b) Consistency adjustments	12	71
c) Appetite	12	71
d) Assistance with feeding (feeding support)	4	24
Instructions on how to assess a patient’s nutritional status	6	35
Algorithm guiding nutritional care options (assessment tool)	9	53
General management, diet and feeding of children and adults with Ebola virus disease		
Advice on energetic needs	10	59
Food products		
a) Recommending use of specialised products^*^	17	100
b) Rationale on use of specialised products^*^	14	82
c) Instructions on what food products to avoid and why	8	47
d) Instructions on how to improve palatability of ORS, CSB etc.	3	18
Specific guidance on how to and what to feed		
a) Infants <6 months	13	76
b) Children 6–23 months	13	76
Instructions on whether or how to use NGT feeding	12	71
Instructions on quantities of food/drinks needed per patient for different age groups		
a) <6 months	9	53
b) 6–23 months	11	65
c) 24–59 months	8	47
d) Older children	8	47
e) Adults	9	53
Instructions on meal and snacks frequency for different age groups	13	76
Information on interactions of certain drugs used on EVD treatment and food/drinks	2	12
Instructions on night-time feeding	5	29
Instructions on whether and how to monitor patient’s food/drink intake	5	29
Intake of specific nutrients		
Advice on need for provision of specific nutrients		
a) Protein	6	35
b) Vitamin A	3	18
c) Vitamin C	1	6
d) Na	1	6
e) K	9	53
f) Ca	2	12
g) Mg	3	18
h) P	2	12
i) Zn	7	41
j) Others (e.g. multivitamins, vitamin K, Fe)	8	47
Symptoms of Ebola virus disease that affect nutritional care and status		
Instructions on how to deal with specific symptoms and dietary intake/needs		
a) Nausea	3	18
b) Vomiting	3	18
c) Diarrhoea	6	35
d) Severe dehydration	7	41
e) Hypoglycaemia	3	18
f) Hypokalaemia	3	18
Food preparation and distribution		
Food preparation		
a) Instructions on preparation of specific food/drinks	11	65
b) Instructions on how to organise food preparation	9	53
c) Instructions on how to organise quality control measures if food preparation is outsourced	1	6
Instructions on how to provide food to individual patients		
a) Packaging	3	18
b) Allocation system	2	12
c) Which utensils	11	65
d) Advice on temperature	4	24
Instructions on specific advice for families if they want to bring meals for a patient	4	24
Instructions on how to organise feeding support		
a) Who gives the feeding support	9	53
b) How to provide feeding support	10	59
c) Physical feeding support aids	5	29
Instructions on how to calculate		
a) Number of meals needed in an ETU	3	18
b) Food/drink supplies for an ETU	3	18
c) Feeding support needs for patients (hardware and human resources)	0	0
Instructions on how to safely transport food from outside and within ETU to patients	2	12
Instructions on how to set up the food system to provide nutritional care for EVD patients	5	29
Instructions on how to handle leftover food and utensils from bed site	11	65
Patient’s preferences		
Instructions on how to assess/accommodate for patient’s dietary preferences	7	41
Instructions on how to obtain patient’s feedback on nutritional care during stay in ETU	3	18
Malnutrition and/or anthropometry		
Instructions on if, when and how to conduct anthropometric measurements	12	71
Instructions on whether or how to adjust treatment for malnourished patient with EVD	4	24
Discharge		
Instructions on discharge procedures and food provision for recovery	11	65
Job instructions		
Job descriptions of various roles in the nutritional care for EVD patients	5	29
Forms		
Patient forms related to nutrition		
a) Assessment	5	29
b) Monitoring	7	41
c) Meal calculations	4	24

EVD, Ebola Virus Disease; ETU, Ebola Treatment Unit; ORS, oral rehydration salt; NGT, naso-gastric tube.

* Ready-to-use therapeutic foods, high-energy biscuits, therapeutic milk, ready-to-use supplementary foods and/or corn soy blend (CSB).

More than half of the guidance materials (59 %) contained clarifications of principles of treatment in relation to nutrition.

### Ebola virus disease and feeding phases/groups

Around two-thirds provided descriptions of different feeding phases during illness (71 %) and feeding groups related to consistency of foods (liquid, semi-solid and sold) (65 %).

### Assessment of patients with Ebola virus disease

Around one-third of materials provided instructions on how to assess patients’ nutritional status, and 71 % advised on patients’ appetite assessment. There were only two documents with instructions on pregnant or lactating women and their nutritional needs. Only four documents (24 %) included assessments on the need for physical feeding support, for the critically ill and/or paediatric cases.

### General management, diet and feeding of children and adults with Ebola virus disease

Fifty-nine percent contained advice on energetic needs of patients in an ETU. All materials recommended the use of specialised products (therapeutic milk, ready-to-use therapeutic food, corn soy blend, etc.), of which 82 % explained some rationale behind the recommendation. Many materials (76 %) had instructions on what to feed children aged 0–23 months and how, and contained instructions on meal and snack frequency for different age groups. However, instructions on quantities of food and drinks needed per patient for each age group were more limited (47–65 %). Seventy-one percent contained instructions on whether to use, and if so, how to use naso-gastric tube feeding for patients in ETU. Older guidance materials developed during the West Africa outbreak were more restrictive on using naso-gastric tube. Eighteen percent had instructions on how to improve the palatability or acceptability of products, for example, oral rehydration salts (ORS), corn soy blend. Almost half of the guidance materials advised to avoid certain products. In some cases, use of sugary carbonated drinks and (fruit) juices were discouraged^(3,18)^. However, advice in favour of use of (fruit) juices was found in some materials from humanitarian organisations. WHO’s pocketbook gave contradictory advice on the same issue^(20)^. Few guidance materials (29 %) contained information on whether and how to monitor patients’ food/drinks intake or instructions on night-time feeding.

### Intake of specific nutrients

Advice on the need for provision of specific nutrients varied and mostly included K (53 %), Zn (41 %), and to some extent, protein (35 %). Less than one-fifth mentioned vitamin A.

### Symptoms of Ebola virus disease that affects nutritional care and status

A few guidance materials (<20 %) gave instructions on how to deal with specific symptoms and dietary intake or needs related to, for example, nausea, vomiting, hypoglycaemia and hypokalaemia. More information was provided on diarrhoea (35 %) and severe dehydration (41 %).

### Food preparation and distribution

Guidance on the operational aspects of food preparation and distribution to patients in an ETU varied. Concerning food preparation, 53–65 % gave some instructions on preparation of specific food/drinks and how to organise it. From previous work, it is known that half of the food preparation was outsourced to catering companies^(2)^, yet only one document contained instructions on how to organise quality control measures related to outsourced food preparation. Except for guidance on which eating or drinking utensils to use, there were limited instructions on how to provide food to individual patients (e.g. packaging, allocation system) or how to deal with meals offered by families (12–24 %). Over half (53–59 %) provided instructions on how to organise feeding support and by whom though no document gave guidance on how to calculate, for example, human resources requirements for this. Limited guidance (<20 %) was given on how to calculate the number of meals and supplies, taking into account the different consistencies of patients’ meals. Only two documents (12 %) gave some instructions on how to safely transport food from outside and within the ETU to the patients. Almost two-thirds provided instructions on leftover food and utensils retrieval from the patients’ bed sites.

### Patient’s preferences

Various materials covered some guidance on how to assess or accommodate patients’ dietary preferences (41 %) or how to obtain feedback on nutritional care from patients within ETU (18 %).

### Malnutrition, anthropometry and discharge

Many materials (71 %) contained guidance on if, when and how to conduct anthropometric measurements; most only advised the use of these measurements at time of discharge. Two-thirds had some instructions on discharge procedures and food provision for recovery for discharged patients.

Guidance materials provided by Save the Children and the Ministry of Health and Social Welfare from Liberia were the most comprehensive, followed by the pocket guide for the frontline health worker from WHO^(10,14,20)^. These materials addressed approximately 60–73 % of the listed topics and provided therefore relatively more guidance on the nutritional care for patients in ETU. There were no substantial differences between older (2014–2017) and more recent (2018–2019) developed guidance materials.

## Discussion

This is the first study to analyse guidance materials on nutritional care for EVD issued since 2014 by non-governmental organisations, the Red Cross/Red Crescent Movement, governments and UN agencies. Though an earlier investigation among practitioners^(2)^ showed the need for increased inclusion of local food products in the patients’ diets, especially for adults, very few of the studied guidance documents stressed the importance of, or instructed on the inclusion of local foods. Similarly, earlier investigation among practitioners also noted absence of guidance on how to organise a food system within an ETU (e.g. how to organise patients’ diets, transfer of food to patients, planning of supplies concerning the number of patients) in the 2014 Interim Guidelines^(2,3)^. While these specific issues were largely absent in the currently reviewed documentation, some governmental, non-governmental organisations and Red Cross/Red Crescent documents included some details related to food preparation and distribution. One aspect, which includes retrieval of left-over food, is particularly important because of the highly infectious environment in the context of EVD. Minimising the volume of contaminated left-over food stresses the importance of accommodating patients’ dietary preferences and feedback on the nutritional care. It is striking that these last two topics were poorly covered in most guidance materials.

Feeding support to very sick and/or paediatric patients in ETU is paramount and instructions on how to assess the individual need was mostly lacking, though guidance on how to provide the support was occasionally included. Most guidance did not mention anthropometric measurements at admission of patients in ETU. Though speculative, this might be due to workload upon admission in an ETU for health care workers and the condition of malnutrition might possibly be perceived as a lower medical priority. Yet, it is widely known that malnutrition as co-morbidity in any infectious disease, including EVD, or in patients who are critically ill, will jeopardise response to treatment^(22–25)^.

Night-time feeding was mentioned in few guidance materials but can be important during an EVD outbreak, especially in warmer climates^(2)^. In the West Africa outbreak, patients were sometimes more alert due to cooler temperatures, and therefore appetite was higher. Eating during night-time contributed to fulfilling the energetic needs, particularly of convalescent patients. Three out of seventeen documents included some advice on how to make, for example, ORS or corn soy blend more palatable or acceptable. This aspect is particularly important for ORS as it might increase the uptake, especially when commercially flavoured ORS is offered^(14,26)^. Guidance on the use of juices and sugary/carbonated drinks was contradictory. However, some practitioners have advocated for their usefulness in increasing fluid intake despite their high osmolarity potentially aggravating diarrhoea^(2,4)^.

Before 2014, limited literature was available on nutritional care of EVD patients. Practitioners encountered difficulties in finding the most appropriate approach^(2,27)^, and organisations and governments developed their own guidance in the absence of a solid scientific base^(2)^. Care for EVD patients is complex because of its highly infectious character, the specific symptoms interfering with patients’ ability to eat, the severity of the pathology, the treatment in relatively low resourced contexts and limited possibilities for relatives to feed the patients^(2)^. This combination requires comprehensive guidance for organisations and governments that provide nutritional care in ETU compared with other diseases. Such guidance should include topics, such as the nutritional assessment of patients with EVD, the general management, diet and feeding of children and adults with EVD, nutrients that need specific attention during treatment, symptoms affecting the patient’s nutritional care and status, the organisation of the food and distribution system in and around an ETU, the organisation of anthropometric measurements, and how to address patients’ dietary preferences.

This analysis shows that substantial guidance materials have been developed covering a wide range of relevant topics. Although every EVD outbreak response must be contextualised because of the complexity of EVD and its case management, it is important to work towards consolidation of the various guidance materials. Therefore, it is essential to resolve potential technical differences (e.g. on use of naso-gastric tube, choice of oral intake of fluids other than ORS) and to provide improved, comprehensive, consistent and practical guidance. The findings of this study include a comprehensive list of topics, which may inform the future revised guidelines from normative UN organisations and governments of countries affected by EVD.

## Limitations

Only thirteen organisations and governments were included in this review; therefore not all guidance materials developed since 2014 were included. However, guidance materials from most major organisations and governments involved in the response were analysed.

## Conclusion

Since 2014, numerous materials have been developed by organisations and governments on nutritional support in ETU for adults and children. Though scientific evidence on the most appropriate nutritional care is still lacking, there is a need for improved, comprehensive, consistent and operational guidance.

## Supplementary material

For supplementary material accompanying this paper visit https://doi.org/10.1017/S136898002000261X. click here to view supplementary material
