# Supplementary material for: Guidance materials from 2014 to 2019 on nutritional care for Ebola patients in Ebola Treatment Units: an analysis
Source: Public Health Nutr. 2020 Aug 20;24(1):139–45. doi: 10.1017/S136898002000261X (PMC7754564; doi:10.1017/S136898002000261X)
Supplement: Supplementary file 1 [file S136898002000261Xsup001.docx]

Nutritional Care Guidance Materials for Ebola Patients in Ebola Treatment Units	Humanitarian Organizations (references 5-12)								Governmental agencies					United Nations Agencies				
	1	2	3	4	5	6	7	8	9	10	11	12	13	14	15	16	17	
	Organization 1	Organization 2	Organization 3	Organization 4	Organization 5	Organization 6	Combined Organizations 3 and 7	Organization 7	Govt Sierra Leone (reference 13)	MoH Liberia (reference 14)	MoH DRC -1 (reference 16)	MoH DRC - 2 (reference 17)	MoH DRC - 3(reference 18)	UNICEF (reference 19)	WHO/ UNICEF/WFP (reference 3)	WHO (reference 20)	WHO (reference 21)	n=17
**Validity of guidance document**																		
Date/year of publication		2014	2015	2015	2015	2015	2016	2018		2014	2018	2018	2018		2014	2016	2019	**14 (82%)**
Contains a statement on duration of validity of document and its recommendations															1			**1 (6%)**
**Purpose of guidance document**																		
Clarification on the principles of treatment of EVD in relation to nutritional care and needs					1		1	1	1	1	1	1	1		1	1		**10 (59%)**
**EVD and feeding phases/groups**																		
Description of disease and feeding phases																		
a) affecting nutritional intake			1		1	1	1	1	1	1	1		1		1	1		**11 (65%)**
b) causing specific nutritional needs					1	1	1		1	1	1		1		1	1	1	**10 (59%)**
c) different feeding phases (maintenance, boost, transitions)	1		1			1	1	1		1	1		1	1	1	1	1	**12 (71%)**
Description of different feeding groups (consistency: solid, semi-solid, liquid)		1	1		1	1	1	1	1	1	1		1		1			**11 (65%)**
**Assessment of patients with Ebola virus disease**																		
Instructions on whether and how to assess a patient's dietary needs																		
a) pregnant and lactating women		1														1		**2 (12%)**
b) consistency adjustments	1	1	1			1	1	1	1		1	1	1		1	1		**12 (71%)**
c) appetite		1	1			1	1	1	1	1	1		1		1	1	1	**12 (71%)**
d) assistance with feeding (feeding support)		1				1	1									1		**4 (24%)**
Instructions on how to assess a patient's nutritional status		1	1									1	1			1	1	**6 (35%)**
Algorithm guiding nutritional care options (assessment tool)			1			1	1	1		1		1	1		1	1		**9 (53%)**
**General management, diet and feeding of children and adults with Ebola virus disease**																		
Advice on caloric needs		1	1			1	1	1	1	1		1			1	1		**10 (59%)**
Food products																		
a) recommending use of specialized products*	1	1	1	1	1	1	1	1	1	1	1	1	1	1	1	1	1	**17 (100%)**
b) rationale on use of specialized products*			1	1	1	1	1	1	1	1	1	1	1		1	1	1	**14 (82%)**
c) instructions on what food products to avoid and why					1	1		1		1		1	1		1	1		**8 (47%)**
d) instructions on how to improve palatability of ORS, CSB etc.			1							1						1		**3 (18%)**
Specific guidance on how to and what to feed																		
a) infants <6 months	1	1	1		1	1			1	1	1	1	1	1		1	1	**13 (76%)**
b) children 6-23 months	1	1	1		1	1			1	1	1	1	1	1		1	1	**13 (76%)**
Instructions on whether or how to use NGT feeding		1	1		1	1	1	1	1	1			1		1	1	1	**12 (71%)**
Instructions on quantities of food/drinks needed per patient for different age groups																		
a) <6 months		1	1		1		1		1	1		1	1			1		**9 (53%)**
b) 6-23 months		1	1		1	1	1	1	1	1		1	1			1		**11 (65%)**
c) 24-59 months		1	1		1	1	1	1		1			1					**8 (47%)**
d) older children		1	1		1	1	1			1			1			1		**8 (47%)**
e) adults		1	1		1	1	1	1		1			1			1		**9 (53%)**
Instructions on meal and snacks frequency for different age groups		1	1	1	1	1	1	1	1	1		1	1		1	1		**13 (76%)**
Information on interactions of certain drugs used for EVD treatment and food/drinks						1				1								**2 (12%)**
Instructions on night-time feeding			1	1		1	1	1										**5 (29%)**
Instructions on whether and how to monitor patient's food/drink intake			1	1		1				1						1		**5 (29%)**
**Intake of specific nutrients**																		
Advice on need for provision of specific nutrients																		
a) protein			1			1	1	1							1	1		**6 (35%)**
b) vitamin A												1	1			1		**3 (18%)**
c) vitamin C				1														**1 (6%)**
d) sodium															1			**1 (6%)**
e) potassium	1					1	1	1	1	1					1	1	1	**9 (53%)**
f) calcium												1			1			**2 (12%)**
g) magnesium									1						1	1		**3 (18%)**
h) phosphorous									1						1			**2 (12%)**
i) zinc				1		1			1	1		1			1	1		**7 (41%)**
j) others (e.g. multivitamins, vitamin K, iron)				1			1	1			1	1			1	1	1	**8 (47%)**
**Symptoms of Ebola virus disease that affect nutritional care and status**																		
Instructions on how to deal with specific symptoms and dietary intake/needs																		
a) nausea					1	1				1								**3 (18%)**
b) vomiting						1										1	1	**3 (18%)**
c) diarrhoea						1	1	1					1		1	1		**6 (35%)**
d) severe dehydration			1			1				1			1		1	1	1	**7 (41%)**
e) hypoglycaemia					1											1	1	**3 (18%)**
f) hypokalaemia						1				1						1		**3 (18%)**
**Food Preparation and Distribution**																		
Food preparation																		
a) Instructions on preparation of specific food/drinks		1	1	1	1	1	1	1	1	1			1		1			**11 (65%)**
b) Instructions on how to organize food preparation		1	1	1		1	1	1	1	1			1					**9 (53%)**
c) instructions on how to organize quality control measures if food preparation is outsourced										1								**1 (6%)**
Instructions on how to provide food to individual patients																		
a) packaging			1			1				1								**3 (18%)**
b) allocation system				1		1												**2 (12%)**
c) which utensils		1	1		1	1	1	1	1	1		1			1	1		**11 (65%)**
d) [illegible]	[illegible]	[illegible]	[illegible]	[illegible]	[illegible]	[illegible]	[illegible]	[illegible]	[illegible]	[illegible]	[illegible]	[illegible]	[illegible]	[illegible]	[illegible]	[illegible]	[illegible]	[illegible]
